# Acidic and Alkaline pH Stresses Impair Tomato Seed Germination and Seedling Growth via Disruption of Reactive Oxygen Species and Auxin Homeostasis

**DOI:** 10.3390/plants15132017

**Published:** 2026-06-29

**Authors:** Huabin Liu, Feiyan Li, Yueyue You, Ailing Chen, Mengjie Li, Jizhou Wang, Qiong Luo, Qinghai Gao

**Affiliations:** 1 College of Biomedicine and Health, Anhui Science and Technology University, Chuzhou 233100, China; lifeiyan@ahstu.edu.cn (F.L.); you@ahstu.edu.cn (Y.Y.); 13866731916@163.com (A.C.); limengjie1@ahstu.edu.cn (M.L.); wangjizhou@ahstu.edu.cn (J.W.); 2 College of Agriculture, Anhui Science and Technology University, Chuzhou 233100, China; 3 College of Life Sciences, Qinghai Normal University, Xining 810008, China; qiongluo812@163.com

**Keywords:** seed germination, seedling growth, pH stress, auxin, ROS, *Solanum lycopersicum*

## Abstract

Soil pH is a critical environmental determinant of seed germination, seedling establishment, and ultimately crop yield. However, the physiological and molecular mechanisms underlying pH stress-mediated inhibition of germination and early seedling development remain poorly understood. Here, tomato was employed as a model system to systematically evaluate the dose-dependent effects of pH stress (ranging from pH 3.5 to 10.5) on germination performance and post-germinative growth. Our results demonstrate that both acidic and alkaline conditions significantly suppressed germination parameters in a pH intensity-dependent manner. Concurrently, seedling growth was markedly inhibited, root and hypocotyl elongation declined progressively, and total seedling biomass decreased substantially. Exposure to acidic (pH 3.5) or alkaline (pH 9.5) stress reduced seed viability and triggered a robust reactive oxygen species (ROS) burst and cell death. Biochemical assays revealed that acidic and alkaline stress disrupted redox homeostasis by compromising the coordinated activity of antioxidant enzymes, elevating membrane lipid peroxidation, and impairing osmotic adjustment capacity. Furthermore, acid and alkaline stress-induced inhibition of root growth coincided with diminished root cell viability and reduced endogenous auxin accumulation. Gene expression analyses showed that acidic and alkaline stress downregulated auxin biosynthesis genes and cell wall-associated genes involved in extension and modification, including *EXPs* and *XTHs*. Notably, IAA priming effectively rescued germination and early seedling growth under alkaline stress. Collectively, these findings elucidate a mechanistic framework linking pH-induced oxidative damage, auxin deficiency, and cell wall remodeling to impaired seed germination and seedling establishment and identify IAA priming as a physiologically grounded strategy to enhance crop resilience in alkali-affected marginal soils.

## 1. Introduction

Soil constitutes one of the most complex and dynamic terrestrial ecosystems. It hosts immense microbial and faunal diversity, underpins terrestrial plant productivity, and serves as the critical interface linking biogeochemical cycles with ecosystem functioning. Plants mediate essential soil processes, including mineral nutrient cycling, organic matter transformation, water retention, and structural stabilization, thereby sustaining ecological balance. Soil pH, a master variable governing soil chemical and biological properties, exerts profound influence on plant growth, species distribution, and community composition. By convention, soils are classified according to pH as acidic (pH < 6.5), neutral (pH 6.5–7.5), or alkaline (pH > 7.5). This pH gradient strongly shapes vegetation patterns across landscapes, with distinct plant taxa exhibiting characteristic pH optima. For instance, *Camellia sinensis*, *Pinus massoniana*, *Rhododendron simsii*, and *Vaccinium* prefer acidic conditions; *Cupressus funebris*, *Beta vulgaris*, *Tamarix chinensis*, and *Elaeagnus angustifolia* are adapted to alkaline soils; whereas *Gossypium hirsutum*, *Hordeum vulgare*, and *Triticum aestivum* prefer near-neutral conditions. Notably, certain species, including sesame and buckwheat, exhibit broad pH tolerance. Such differential adaptation arises primarily from long-term evolutionary selection and local adaptation to native soil conditions [1,2,3]. Although extreme pH adaptation in plants is of great agronomic and ecological significance, its underlying molecular and physiological bases remain largely uncharacterized, thereby limiting advances in breeding tolerant crops for acid- or alkali-stressed marginal lands.

Soil pH fluctuations alter the solubility and mobility of mineral elements, thereby modulating nutrient uptake and utilization by roots. An optimal rhizosphere pH facilitates root growth, nutrient acquisition, and physiological metabolism in plants. In contrast, extreme pH conditions, either strongly acidic or alkaline, trigger a suite of stress symptoms, including micronutrient deprivation, macronutrient imbalance, and perturbations in redox homeostasis and energy metabolism. Under alkaline stress, high ambient pH coupled with elevated osmotic pressure impairs cell membrane integrity and disturbs intracellular ion homeostasis, especially the compartmentalization of K^+^ and Ca^2+^, ultimately compromising cellular physiological function and plant growth [4,5]. Conversely, soil acidification mobilizes phytotoxic metals, including Al^3+^, Mn^2+^, and Cd^2+^, elevating their rhizosphere bioavailability and inducing metal toxicity, which damages root meristems, inhibits root elongation, and hinders seedling establishment [6,7]. In addition, acidic or alkaline stress triggers a burst of ROS and disturbs antioxidant enzyme activities, which in turn causes ROS overaccumulation and severe membrane lipid peroxidation, ultimately restraining plant growth [8,9,10]. Critically, regional soil pH heterogeneity is frequently coupled with secondary abiotic stresses. Aluminum toxicity predominates in strongly acidic soils, while combined salt-alkali stress prevails in arid and semi-arid alkaline regions, synergistically exacerbating plant growth inhibition [11,12]. Although the multifaceted impacts of pH stress have been increasingly acknowledged, the integrated physiological and molecular mechanisms governing seed germination and early seedling establishment under pH stress remain poorly understood.

Seed germination is a tightly coordinated developmental process encompassing imbibition, activation of hydrolytic enzymes, mobilization and metabolic utilization of stored reserves, and radicle emergence through the seed coat. As enzymatic activity and cellular osmotic homeostasis are highly sensitive to pH perturbations, these sequential physiological and biochemical events exhibit pronounced pH dependence. For most plant species, germination rates peak under near-neutral pH conditions. In contrast, both acidic and alkaline pH conditions suppress seed germination in a dose-dependent manner—increasing acidity or alkalinity correlates with progressive declines in germination percentage, speed, and uniformity. Notably, interspecific variation in pH tolerance is well documented: seeds of *Camellia sinensis* and *Pinus massoniana* are adapted to acidic substrates, whereas those of *Beta vulgaris* and *Tamarix chinensis* exhibit superior germination performance under alkaline conditions. By comparison, *Triticum aestivum* and *Zea mays* display optimal germination within the near-neutral range. These species-specific germination optima reflect evolutionary adaptations to native soil pH regimes, underpinning ecological niche partitioning and agricultural suitability [1,2,3]. Critically, reduced seed germination and seedling emergence in acidified or alkalized soils directly constrain crop establishment, contributing to yield loss, quality deterioration, and narrowing of cultivar options. Currently, enhancing seed germination efficiency and early seedling vigor represents a primary agronomic strategy for mitigating acid-base stress. However, the integrated physiological and molecular mechanisms regulating seed germination and post-germinative seedling establishment under pH stress remain poorly resolved, which limits the development of crop cultivars adapted to acidified or alkalized soils.

Phytohormones, particularly auxin, play a central role in regulating multiple developmental transitions throughout the plant life cycle, including seed development, dormancy induction and release, vitality, germination, post-germinative seedling morphogenesis, and vegetative growth. Auxin also modulates plant responses to diverse abiotic stresses, including salinity-alkalinity [13,14], aluminum toxicity under acidic conditions [15,16], heavy metal exposure [17,18], and temperature extremes [19,20]. Critically, auxin signaling operates in a tightly dose-dependent manner: optimal concentrations promote germination competence and radicle emergence, whereas supra-optimal levels reinforce dormancy maintenance and suppress germination initiation [21]. Genetic evidence corroborates this biphasic regulation: loss-of-function mutants defective in auxin biosynthesis exhibit earlier germination and reduced dormancy, while gain-of-function lines or exogenous application of high-dose auxin intensifies dormancy and delays germination [21]. Auxins modulate rice adaptation to alkaline stress through coordinated regulation of ROS homeostasis and root system architecture [14]. Rice genotypes with elevated endogenous IAA levels demonstrate enhanced ROS-scavenging capacity, increased root biomass, and superior alkaline stress tolerance relative to low-IAA counterparts [14]. Consistently, exogenous IAA application augments ROS detoxification and promotes primary and lateral root elongation, thereby improving alkaline stress resilience in rice [14]. Complementary evidence from *Arabidopsis thaliana* indicates that mild alkalinity (pH 7.5) stimulates primary root growth via auxin-mediated signaling pathways [22]. In contrast, auxin dynamics under acidic-aluminum stress exhibit species- and tissue-specific complexity: in maize, Al^3+^ triggers rapid auxin efflux from root tips via ZmPGP1-mediated transport, depleting local IAA pools and inhibiting root elongation [23]. Conversely, in Arabidopsis, Al^3+^ disrupts PIN2 endocytosis and increases PIN protein abundance, causing auxin accumulation in the roots and consequent growth arrest [24,25]. In tea plants, the UDP-glucosyltransferase CsUGT84J2 promotes the growth of plants in acid-aluminum soils by regulating auxin homeostasis [15]. Although the functions of auxin have been characterized in many plant species, the factors underlying these differences in the regulation mechanism of auxin remain unclear. Given auxin’s pivotal role in integrating developmental cues with environmental signals, perturbations in its biosynthesis, transport, or perception invariably compromise plant growth, development, and stress resilience. Although reports have highlighted the potential of auxin in regulating plant stress tolerance, given that tomatoes are one of the most widely cultivated horticultural crops worldwide, elucidating the physiological adaptation mechanisms underlying germination and seedling growth under pH gradients is crucial for tomato cultivation in acidic and alkaline soil regions and for improving tomato yields.

To elucidate the regulatory mechanism underlying tomato responses to pH stress, we systematically characterized germination performance and early seedling establishment across a broad pH gradient (pH 3.5–10.5). Concurrently, we evaluated the efficacy of IAA seed priming in mitigating pH-induced inhibition of seed germination and seedling growth. Furthermore, we also focused specifically on ROS accumulation, antioxidant homeostasis, cell viability, proline levels, and auxin responses to elucidate the physiological responses and auxin regulatory mechanisms in plants under acidic and alkaline conditions.

## 2. Results

### 2.1. Acidic and Alkaline Stresses Inhibit Tomato Seed Germination and Seedling Growth and Disrupt Physiological Metabolism

**Figure 1:**
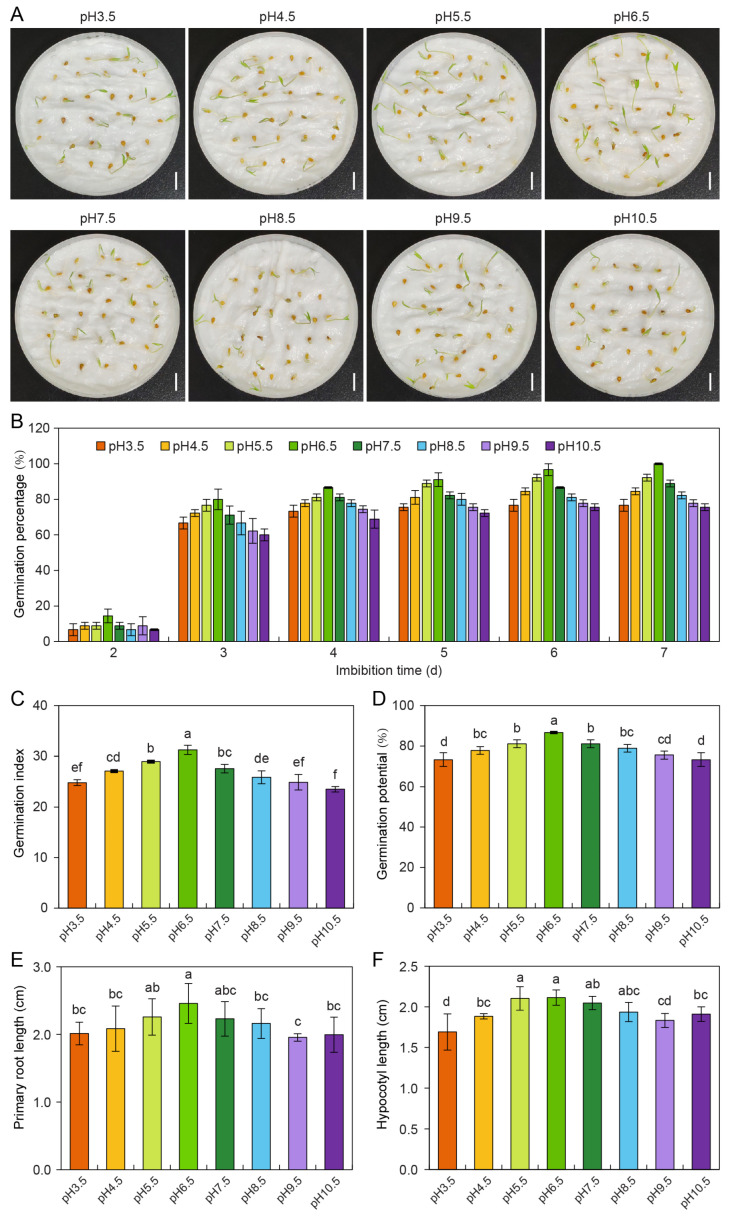
Tomato seed germination and seedling growth across a pH gradient. (**A**) Phenotypes of tomato seeds after 4 days of germination at different pH levels (pH 3.5, 4.5, 5.5, 6.5, 7.5, 8.5, 9.5, and 10.5). Bars = 1 cm. (**B**–**D**) Time course of seed germination (**B**), germination index (**C**), and germination potential (**D**) under varying pH conditions. (**E**,**F**) Primary root length (**E**) and hypocotyl length (**F**) of tomato seedlings at 7 days post-germination under pH gradient conditions. Group differences were assessed by ANOVA coupled with Duncan’s test. Values marked with different letters are significantly different (*p* < 0.05).

To evaluate the effects of acidic and alkali stresses on tomato seed germination, we measured the germination rate, germination vigor, and germination index of tomato seeds across a pH gradient (pH 3.5–10.5) (Figure 1). Under near-neutral pH conditions (pH 6.5), tomato seed germination rate, germination potential, and germination index all attained their maximum values (Figure 1A–D). Under acidic conditions (pH < 6.5), these three germination parameters declined progressively with decreasing pH (Figure 1A–D). Similarly, under alkaline conditions (pH > 7.5), a dose-dependent inhibitory effect was observed: all three parameters decreased gradually with increasing pH (Figure 1A–D). Since germination efficiency directly determines subsequent seedling morphogenesis and vigor, we further assessed the impact of pH stress on early seedling establishment by measuring hypocotyl and root length, lateral root number, and fresh biomass (Figure 1E,F and Figure S1). Both acidic (pH < 6.5) and alkaline (pH > 7.5) conditions significantly suppressed hypocotyl and primary root elongation and reduced total seedling fresh weight (Figure 1E,F and Figure S1). Notably, severe acidification (pH 3.5) markedly impaired lateral root initiation and branching architecture (Figure S1B).

Plant growth and development depend critically on both root-mediated nutrient acquisition from the soil and leaf-based photosynthetic energy production. Photosynthetic pigments are essential for light capture and photoprotection. Compared with the near-neutral control (pH 6.5), total chlorophyll content in tomato seedling leaves declined significantly under severe acidic conditions (pH 3.5), whereas both total chlorophyll and carotenoid contents were markedly reduced under strong alkaline stress (pH 9.5) (Figure S1). Notably, acidic stress preferentially diminished chlorophyll a and chlorophyll b levels, while alkaline stress exerted a stronger suppressive effect on chlorophyll a and carotenoids. This pigment degradation reflects impaired photosynthetic capacity and reduced carbon assimilation efficiency, which is consistent with the observed suppression of seedling biomass accumulation under both acidic (pH 3.5) and alkaline (pH 9.5) conditions.

**Figure 2:**
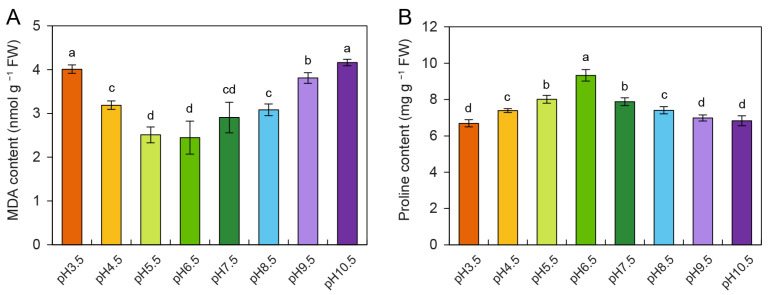
MDA and proline concentrations in tomato seedlings under pH gradient conditions. (**A**,**B**) MDA (**A**) and proline (**B**) concentrations in tomato seedlings after 7 days of exposure to pH gradient conditions (pH 3.5, 4.5, 5.5, 6.5, 7.5, 8.5, 9.5, and 10.5). Group differences were assessed by ANOVA coupled with Duncan’s test. Values marked with different letters are significantly different (*p* < 0.05).

Plants routinely encounter diverse environmental stresses throughout their life cycle, triggering ROS burst and consequent oxidative damage to cell structures. Malondialdehyde (MDA) serves as a biomarker for membrane lipid peroxidation; elevated MDA concentrations directly reflect the extent of membrane integrity loss and functional impairment. To quantify oxidative damage induced by pH stress, we measured MDA content in seedlings across a pH gradient (pH 3.5–10.5) (Figure 2). Under acidic conditions (pH < 6.5), MDA levels increased progressively with decreasing pH (Figure 2A). Under alkaline conditions (pH > 7.5), MDA levels likewise rose significantly with increasing pH (Figure 2A). The minimal MDA accumulation was observed at near-neutral pH (6.5) (Figure 2A), indicating optimal membrane stability. Collectively, these data demonstrate a dose-dependent exacerbation of oxidative membrane damage under both acidic and alkaline stress regimes.

As a major compatible osmolyte in plant cells with potent non-enzymatic antioxidant activity, proline plays a central role in mitigating both abiotic stress and its associated oxidative damage. To assess the impact of pH stress on osmoprotective capacity, we quantified proline accumulation in tomato seedlings across a pH gradient (pH 3.5–10.5) (Figure 2B). Under acidic conditions (pH < 6.5), proline content declined progressively with decreasing pH (Figure 2B). Under alkaline conditions (pH > 7.5), proline content likewise decreased significantly with increasing pH (Figure 2B). Maximum proline accumulation occurred at near-neutral pH (6.5) (Figure 2B), indicating optimal osmotic homeostasis. These findings demonstrate that escalating pH deviation from neutrality impairs cellular osmoprotective capacity in a dose-dependent manner.

### 2.2. Both Acidic and Alkaline Stress Decrease Vitality in Tomato Seeds and Seedling Roots

**Figure 3:**
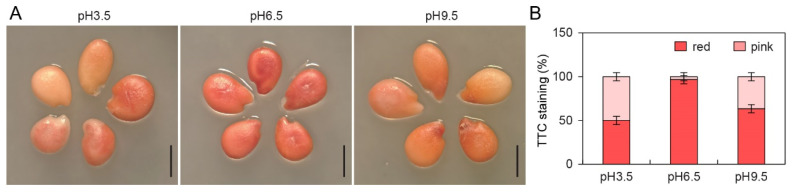
Effects of acid or alkaline stress on tomato seed viability. (**A**,**B**) Viable seed percentages in tomato seeds exposed to acidic (pH 3.5), alkaline (pH 9.5), or control (pH 6.5) conditions were determined by TTC staining. Seed viability is indicated by pigment intensity: red staining denotes high metabolic activity, whereas pale pink staining indicates reduced viability. Bars = 100 µm.

To further evaluate the effects of pH stress on seed viability, TTC staining was employed to evaluate seed vigor. The results showed that tomato seeds under near-neutral pH conditions (pH 6.5) stained deep red, indicating high seed vigor (Figure 3A). In comparison, seeds subjected to acidic (pH 3.5) or alkaline (pH 9.5) treatments exhibited a marked reduction in staining intensity, reflecting low seed vigor (Figure 3A). Statistical analysis demonstrated that approximately 50% of acid-stressed seeds and 37% of alkaline-stressed seeds showed only faint pink staining (Figure 3B). Collectively, these findings indicate that both acidic and alkaline pH stress significantly compromise tomato seed viability.

**Figure 4:**
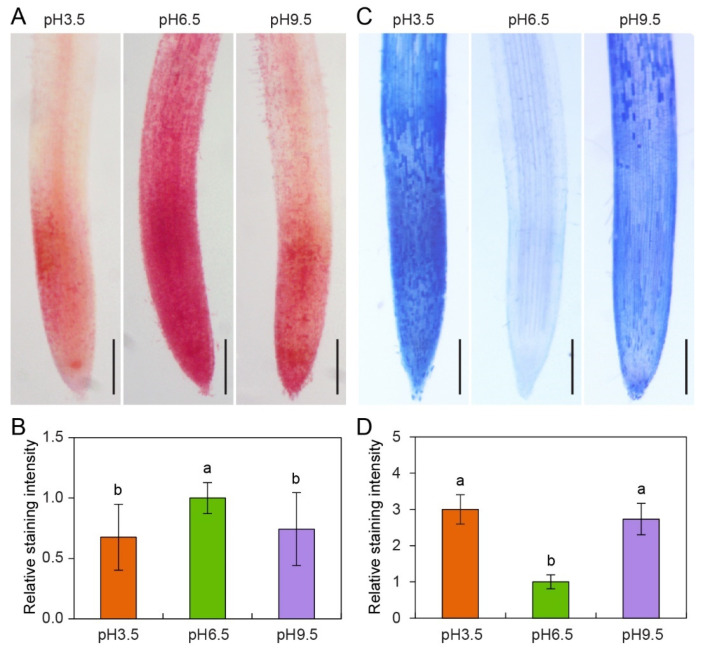
Effects of acid or alkaline stress on cell viability in tomato seedling roots. Three-day-old tomato seedlings were exposed to acidic (pH 3.5), alkaline (pH 9.5), or control (pH 6.5) conditions. (**A**–**D**) Cell viability (**A**,**B**) and cell death (**C**,**D**) were assessed using TTC staining and Evans blue staining, respectively. Group differences were assessed by ANOVA coupled with Duncan’s test. Values marked with different letters are significantly different (*p* < 0.05). Bars = 200 µm.

Root vitality is used to assess the physiological function and environmental adaptability of roots. Phenotypic analysis confirmed that both acidic and alkaline pH stress significantly inhibit primary root elongation in tomato seedlings. To evaluate root vitality, TTC staining was performed to assess root cell activity. Under near-neutral pH conditions (pH 6.5), the root meristematic tissues of tomato seedlings were stained deep red, indicating strong root vitality (Figure 4A,B). In contrast, tomato seedling roots exposed to acidic (pH 3.5) or alkaline (pH 9.5) conditions showed markedly diminished staining intensity (Figure 4A,B), indicating substantial impairment of root vitality.

To further investigate the impacts of acidic and alkaline stress on root vitality, Evans blue and trypan blue staining were applied to assess cell viability. Consistent with the TTC staining results, the root meristematic tissues of tomato seedlings treated with near-neutral pH (pH 6.5) displayed faint staining, indicative of intact root cell vitality (Figure 4C,D and Figure S2). In contrast, seedling roots exposed to acidic (pH 3.5) or alkaline (pH 9.5) stress showed much darker staining, which reflected severe damage to root cell membranes and extensive cell death (Figure 4C,D and Figure S2).

### 2.3. Acidic and Alkaline pH Stress Induce ROS Bursts and Disrupt Homeostasis of the Antioxidant System

**Figure 5:**
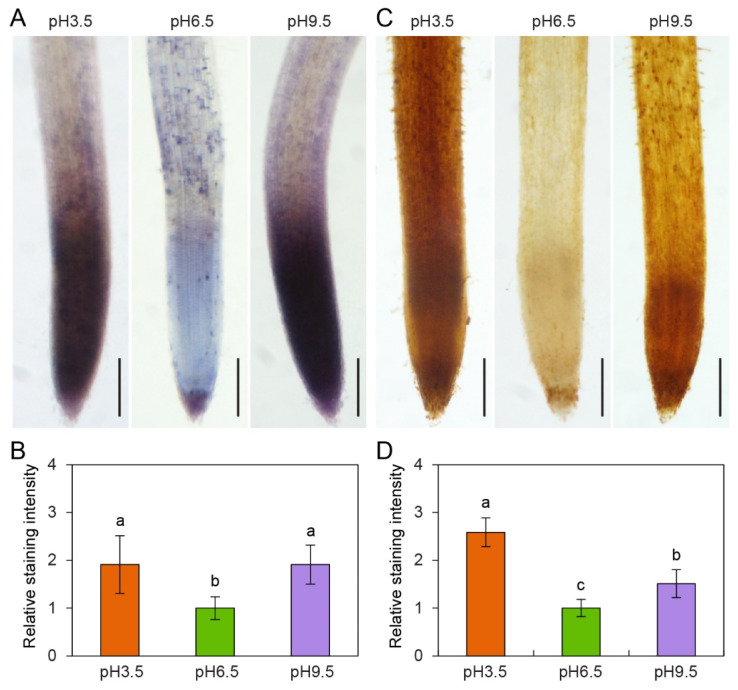
ROS accumulation in tomato seedling roots under acidic or alkaline conditions. Tomato seedlings were exposed to acidic (pH 3.5), alkaline (pH 9.5), or control (pH 6.5) conditions for 6 h. (**A**–**D**) O_2_^·−^ (**A**,**B**) and H_2_O_2_ (**C**,**D**) accumulation was detected using NBT and DAB staining, respectively. Group differences were assessed by ANOVA coupled with Duncan’s test. Values marked with different letters are significantly different (*p* < 0.05). Bars = 200 µm.

ROS, including superoxide anions (O_2_^∙−^) and hydrogen peroxide (H_2_O_2_), function as essential redox signaling molecules in plant abiotic stress responses. They regulate stress adaptation by activating downstream signaling pathways and inducing the transcription of antioxidant and stress-responsive genes. However, prolonged or severe adversity disrupts cellular redox homeostasis, leading to uncontrolled ROS accumulation. Excessive ROS induces oxidative stress, resulting in lipid peroxidation, protein carbonylation, and nucleic acid damage, thereby amplifying the inhibitory effect of pH stress on root elongation. To spatially resolve ROS dynamics under acidic and alkaline pH conditions, we applied NBT staining for O_2_^∙−^ detection and DAB histochemistry for H_2_O_2_ localization in tomato seedling roots. It was found that both acidic (pH 3.5) and alkaline (pH 9.5) stresses led to O_2_^∙−^ and H_2_O_2_ accumulation in the roots (Figure 5). Notably, the accumulation of H_2_O_2_ induced by acidic (pH 3.5) stress was more significant, and the entire roots were stained dark brown (Figure 5C). The H_2_O_2_ burst induced by alkaline (pH 9.5) stress occurred primarily in the meristematic zone and root cap (Figure 5C). Compared with the control (pH 6.5), O_2_^∙−^ bursts under acidic (pH 3.5) and alkaline (pH 9.5) stress occurred in overlapping root zones, predominantly the elongation zone, meristematic tissue, and root cap, indicating shared sites of oxidative initiation (Figure 5A,B). These spatially similar ROS accumulation patterns correlate directly with elevated levels of MDA, confirming that both acidic and alkaline pH stress induce oxidative damage to plasma membranes of tomato seedling roots.

**Figure 6:**
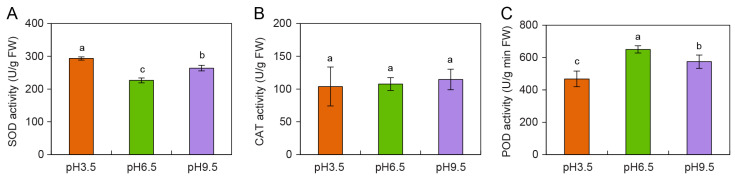
Antioxidant enzyme activity in tomato seedlings under acidic or alkaline conditions. (**A**–**C**) SOD (**A**), CAT (**B**), and POD (**C**) activities of tomato seedlings at 7 days post-germination under acidic (pH 3.5), alkaline (pH 9.5), or control (pH 6.5) conditions. Group differences were assessed by ANOVA coupled with Duncan’s test. Values marked with different letters are significantly different (*p* < 0.05).

Cellular ROS homeostasis is dynamically maintained by the balance between enzymatic generation (primarily via NADPH oxidases and electron transport chains) and enzymatic scavenging through the antioxidant system. This system encompasses three core antioxidant enzymes: superoxide dismutase (SOD), responsible for catalyzing the dismutation of O_2_^∙−^ to H_2_O_2_, while catalase (CAT) and peroxidase (POD) act in concert to detoxify the generated H_2_O_2_. To elucidate the mechanisms by which acidic and alkaline pH stress disturbs ROS homeostasis, we quantified antioxidant enzyme activities in seedlings under pH 3.5, 6.5, and 9.5 conditions. Under both pH 3.5 and pH 9.5 treatments, SOD activity increased significantly, whereas POD activity was markedly suppressed (Figure 6A,C). In contrast, CAT activity remained statistically unchanged across all treatments (Figure 6B). Collectively, these data demonstrate that acidic and alkaline pH stress selectively impair the peroxidase-mediated H_2_O_2_ detoxification pathway while inducing compensatory SOD upregulation, thereby disrupting redox homeostasis and exacerbating oxidative stress.

### 2.4. Acidic and Alkaline pH Stress Affect Auxin Biosynthesis and Accumulation in Tomato

**Figure 7:**
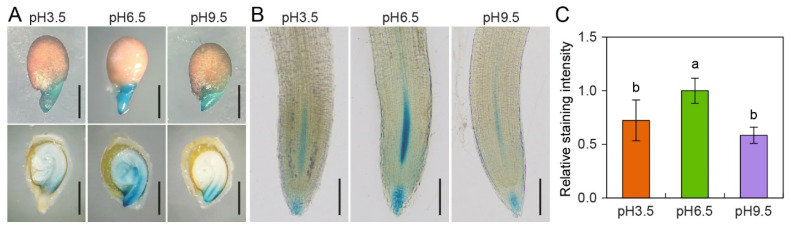
Auxin response in tomato seeds and seedlings under acidic or alkaline conditions. (**A**) Tomato seeds (*DR5:GUS*) were germinated in acidic (pH 3.5), alkaline (pH 9.5), or control (pH 6.5) conditions for 72 h. The upper panel shows DR5:GUS activity in the radicle; the lower panel presents longitudinal sections revealing tissue-specific expression patterns. At pH 6.5, strong DR5:GUS activity is detected in both cotyledons and the radicle; by contrast, expression is markedly diminished in cotyledons and the radicle under acidic and alkaline pH conditions. Bars: 100 µm. (**B**,**C**) GUS histochemical staining (**B**) and quantitative analysis of staining intensity (**C**) in three-day-old tomato seedlings (*DR5:GUS*) following 48 h exposure to acidic (pH 3.5), alkaline (pH 9.5), or control (pH 6.5) conditions. Group differences were assessed by ANOVA coupled with Duncan’s test. Values marked with different letters are significantly different (*p* < 0.05). Bars: 200 µm.

Auxin is a central phytohormone governing seed germination, root meristem maintenance, and primary root elongation. To test whether pH stress alters auxin signaling during early development, we used the auxin-responsive reporter *DR5:GUS* to analyze its activity in tomato seeds and seedling primary roots under pH 3.5, 6.5, and 9.5 conditions. Histochemical GUS staining revealed significantly diminished auxin-responsive signal intensity in seeds, radicles, and the apical region of primary roots under both acidic (pH 3.5) and alkaline (pH 9.5) treatments relative to the control (pH 6.5) (Figure 7). These findings demonstrate that acidic and alkaline pH stress suppress auxin accumulation and/or response in critical growth zones, thereby impairing auxin-dependent developmental programs essential for seed germination and post-germinative root establishment.

**Figure 8:**
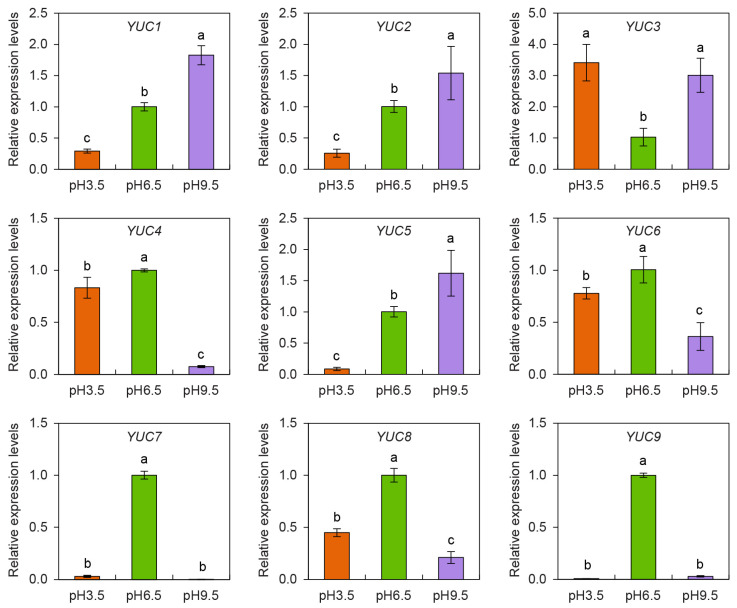
Expression patterns of *YUCs* in tomato seedlings under acidic or alkaline conditions. Tomato seedlings at 7 days post-germination under acidic (pH 3.5), alkaline (pH 9.5), or control (pH 6.5) conditions. Expression pattern of *YUCs* in tomato seedlings was analyzed by qRT-PCR. Group differences were assessed by ANOVA coupled with Duncan’s test. Values marked with different letters are significantly different (*p* < 0.05).

YUCCA (YUC) flavin monooxygenases constitute the rate-limiting enzymatic step in the dominant tryptophan-dependent auxin biosynthesis pathway in plants. To dissect the transcriptional regulation of auxin synthesis under pH stress, we quantified the expression levels of all nine *YUC* paralogs (*YUC1*–*YUC9*) in tomato seedling roots exposed to acidic (pH 3.5), near-neutral (pH 6.5), and alkaline (pH 9.5) conditions. Expression profiling revealed three distinct regulatory patterns: (i) *YUC1*, *YUC2*, and *YUC5* were significantly downregulated by acidic stress but upregulated by alkaline stress; (ii) *YUC4*, *YUC6*, *YUC7*, *YUC8*, and *YUC9* were robustly suppressed under both pH extremes; and (iii) *YUC3* was uniquely induced by both acidic and alkaline treatments, which exhibiting the highest fold-change among all paralogs (Figure 8). Notably, *YUC7* and *YUC9* displayed the most pronounced transcriptional repression in tomato seedling roots under both pH 3.5 and 9.5 conditions, consistent with their established roles in root-specific auxin biosynthesis [16,26]. Collectively, these data demonstrate that acidic and alkaline pH stress suppress the expression of root-predominant *YUC* isoforms, thereby attenuating local auxin biosynthesis—a mechanism directly responsible for the reduced auxin accumulation observed in roots.

### 2.5. Acidic and Alkaline pH Stress Downregulates Cell Wall Modification-Related Gene Expression

**Figure 9:**
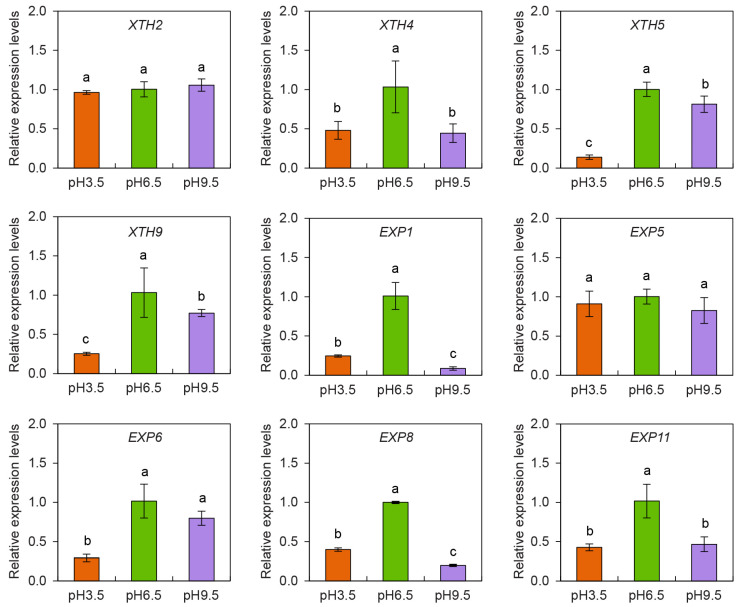
Expression patterns of cell wall modification-related genes in tomato seedlings under acidic or alkaline conditions. Tomato seedlings at 7 days post-germination under acidic (pH 3.5), alkaline (pH 9.5), or control (pH 6.5) conditions. Expression patterns of *XTHs* and *EXPs* in tomato seedlings were analyzed by qRT-PCR. Group differences were assessed by ANOVA coupled with Duncan’s test. Values marked with different letters are significantly different (*p* < 0.05).

Cell wall remodeling is a tightly regulated process governed by families of structural and enzymatic proteins that collectively mediate cellular expansion, tissue differentiation, and organ morphogenesis. Key molecular regulators include expansins (EXPs) and xyloglucan endotransglucosylase/hydrolases (XTHs), both of which are indispensable for seed germination, root architecture formation, and plant growth. To elucidate how acidic and alkaline pH stress disrupts cell wall homeostasis and consequently restricts seedling development, we performed a targeted transcript analysis of genes encoding cell wall-loosening and cell wall-modifying proteins in tomato seedling roots. Plant cell growth and elongation are mechanically constrained by the rigid cell wall. EXPs are a family of non-enzymatic, cell wall-loosening proteins that facilitate wall relaxation and irreversible extension, thereby enabling cellular expansion. Quantitative gene expression analysis revealed that acidic stress significantly downregulated *EXP1*, *EXP6*, *EXP8*, and *EXP11* transcripts, whereas alkaline stress induced significant downregulation of *EXP1*, *EXP8*, and *EXP11* (Figure 9). In contrast, neither acidic nor alkaline stress elicited significant changes in *EXP5* expression levels (Figure 9).

XTHs are key cell wall-modifying proteins that dynamically regulate xyloglucan network architecture through cleavage, transglycosylation, and reintegration, thereby modulating wall mechanics during plant cellular expansion. Exposure to acidic or alkaline pH stress resulted in a significant downregulation of *XTH4*, *XTH5*, and *XTH9* transcript levels, whereas *XTH2* expression was not altered under the same conditions (Figure 9). Collectively, these findings indicate that acid-base stress impairs cell wall extensibility via selective transcriptional repression of specific *XTH* genes, ultimately constraining cellular expansion and inhibiting seedling growth.

### 2.6. Exogenous IAA Mitigates Acid-Base Stress-Induced Inhibition of Tomato Seed Germination and Seedling Growth

**Figure 10:**
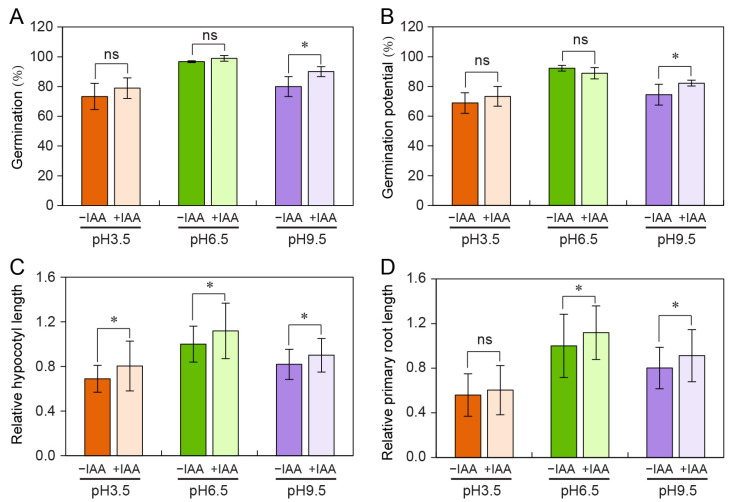
IAA seed priming enhances seed germination and seedling growth under acidic or alkaline conditions. (**A**,**B**) The germination rate (**A**) and germination potential (**B**) of the tomato seeds. (**C**,**D**) Relative hypocotyl (**C**) and root (**D**) length of tomato seedlings. Tomato seeds were pretreated with 10 µM IAA and germinated under pH 3.5, 6.5, and 9.5 conditions for 7 days. The values in the pH 6.5 conditions (without IAA) are set to 1. Data are means ± SD. Asterisks indicate that values between IAA treatments at each pH level differ significantly (* *p* < 0.05). ns represents not significant.

The preceding experiments collectively indicate that auxin signaling is integral to tomato plant responses to both acidic and alkaline stress. To assess the functional role of exogenous IAA in modulating seed germination and seedling growth under pH stress, we conducted seed priming assays via IAA solution soaking. Under alkaline stress, IAA priming significantly enhanced germination rate and germination potential relative to untreated controls (Figure 10). Moreover, IAA treatment markedly promoted seedling growth under alkaline conditions, including root elongation and hypocotyl extension (Figure 10C,D). These data demonstrate that IAA application confers enhanced alkaline stress tolerance during tomato seed germination and seedling establishment. In contrast, while IAA priming elicited a positive trend in germination under acidic stress, the improvement failed to attain statistical significance (Figure 10). Collectively, these results suggest divergent physiological sensitivities and regulatory efficacies of IAA in mediating tomato responses to acidic versus alkaline stress.

**Figure 11:**
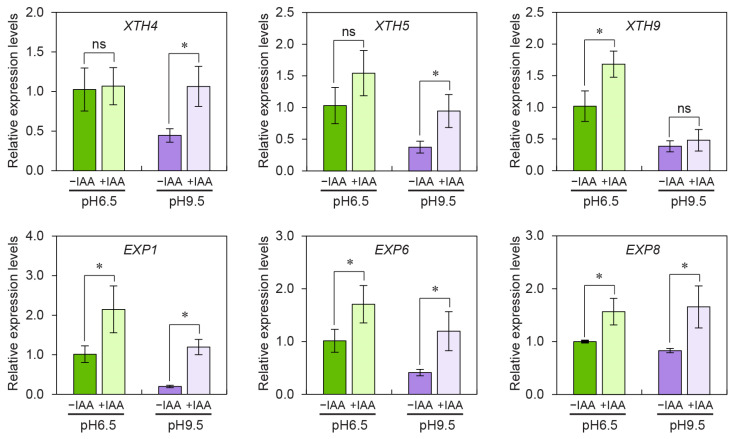
IAA regulated the expression of *XTHs* and *EXPs* under alkaline conditions. Tomato seeds were pretreated with 10 µM IAA and germinated under pH 3.5, 6.5, and 9.5 conditions for 5 days. Expression pattern of *XTHs* and *EXPs* in tomato seedlings was analyzed by qRT-PCR. Asterisks indicate that values between IAA treatments at each pH level differ significantly (* *p* < 0.05). ns represents not significant.

To elucidate the mechanism underlying IAA-mediated alleviation of growth inhibition in tomato seedlings under alkaline pH stress, ROS levels and antioxidant enzyme activities in IAA-treated seedlings were analyzed. Biochemical measurements confirmed that exogenous IAA substantially inhibited ROS overaccumulation under alkaline conditions (Figure S3). Enzyme activity assays further illustrated that IAA distinctly elevated POD activity, while SOD and CAT activities exhibited no obvious changes (Figure S4). Furthermore, qRT-PCR analysis showed that IAA markedly upregulated the expression of cell wall remodeling genes, including *XTH4*, *XTH5*, *EXP1*, *EXP6*, and *EXP8* under alkaline pH stress (Figure 11). Collectively, these findings indicate that IAA pretreatment mitigates alkaline stress-induced growth inhibition in tomato seedlings through dual regulatory actions: attenuation of oxidative stress via selective enhancement of POD activity and promotion of cell growth via transcriptional activation of *XTHs* and *EXPs*.

## 3. Discussion

Soil acidification and alkalization represent two major abiotic stressors threatening global agricultural sustainability. These processes disrupt soil biogeochemical cycles by altering the speciation and bioavailability of essential mineral nutrients, while simultaneously promoting nutrient leaching and mobilizing toxic heavy metals, thereby accelerating soil degradation and imposing severe environmental stress on agroecosystems. The pivotal role of soil pH in sustaining soil health and crop productivity is well established: analogous to core body temperature in homeothermic organisms, soil pH serves as a master regulator of physiological and biochemical processes underpinning soil vitality. As a primary determinant of soil chemical equilibrium, physical structure, and nutrient acquisition efficiency, pH critically governs agricultural soil functionality. Consequently, extreme pH deviations, whether from progressive acidification or alkalization, synergistically interact with declining soil fertility to impair plant growth, development, and yield formation.

Although many studies have proposed maintaining soil pH within a range suitable for plant growth by modifying soil acidification and alkalization [6,27], accelerating climate change and intensified industrial activity are driving unprecedented spatiotemporal variability in soil pH. This variability arises from altered precipitation regimes, elevated ambient temperatures, and widespread agrochemical inputs, including synthetic fertilizers and pesticides. Globally, acidic soils occupy over 40% of arable and potentially arable land, whereas alkaline soils cover approximately 10%; given the immense spatial extent and socioeconomic constraints, large-scale, uniform soil remediation remains impractical. Consequently, elucidating the molecular and physiological framework underlying acidic and alkaline pH stress-mediated inhibition of tomato seed germination and early seedling development, alongside identifying conserved regulatory nodes that orchestrate plant adaptive responses to soil pH fluctuations, represents a strategically robust approach to enhancing crop resilience to edaphic stress. Here, our results reveal that exposure to either acidic or alkaline pH stress markedly suppresses tomato seed germination, as supported by significant decreases in three core germination parameters: germination rate, germination potential, and germination index (Figure 1). Seed viability assays demonstrate that both acidic (pH 3.5) and alkaline (pH 9.5) stress impair cell viability, resulting in insufficient germination momentum and a decreased germination rate (Figure 1 and Figure 3). Furthermore, auxin response signaling assays and longitudinal seed dissection experiments revealed that auxin signaling in the radicle was significantly attenuated under both pH 3.5 and pH 9.5 conditions (Figure 7A). Auxin is considered one of the key hormones involved in regulating seed germination and vitality [21,28]. Some evidence demonstrates that auxin, acting synergistically with abscisic acid (ABA), promotes seed dormancy maintenance while concurrently suppressing germination initiation [21,29]. ABA and gibberellin (GA) are well-established antagonistic phytohormones that govern the developmental switch from seed dormancy to germination. Elevated ABA levels coupled with suppressed GA biosynthesis or signaling promote dormancy maintenance, whereas reduced ABA accumulation alongside enhanced GA activity triggers germination initiation [30]. The increasing evidence demonstrates that auxin exerts a biphasic, concentration-dependent effect on seed germination: low physiological concentrations promote germination, whereas supra-optimal concentrations inhibit it. Critically, the threshold concentration defining this biphasic response is species-specific [31,32]. Collectively, these findings underscore that seed germination competence is coordinately regulated by the interplay among multiple phytohormones, not by any individual hormone or its absolute concentration.

Although exogenous application of phytohormones or chemical priming agents has been shown to markedly improve seed germination, seedling establishment, and abiotic stress tolerance in multiple crop species [33,34,35], the molecular and physiological mechanisms by which auxin-mediated seed priming modulates tomato resilience to abiotic stress remain poorly defined. Our experimental results demonstrate that endogenous auxin levels are significantly depleted during tomato seed germination under acidic (pH 3.5) or alkaline (pH 9.5) stress conditions (Figure 7). In contrast, exogenous application of IAA markedly enhances germination performance under both pH stresses, as evidenced by significant increases in germination rate (Figure 10). Collectively, these findings indicate that IAA seed priming effectively compensates for stress-induced auxin deficiency, thereby restoring physiological auxin homeostasis and promoting robust germination.

Multiple studies have documented that alkaline stress impairs root system architecture and function in plants, manifesting as suppressed adventitious root formation, reduced primary root elongation, and diminished root metabolic activity [9,14,36]. Collectively, these structural and physiological impairments compromise root nutrient and water uptake capacity, thereby constraining overall plant growth and development. Consistent with this, our experiments demonstrate that both acidic (pH 3.5) and alkaline (pH 9.5) stress severely inhibit tomato seedling establishment, as evidenced by significant reductions in fresh biomass, primary root length, and hypocotyl length (Figure 1 and Figure S1). Furthermore, histological and viability assays reveal that pH stress induces progressive loss of cell viability in the root meristematic and elongation zones and triggers programmed cell death specifically in the root tip region (Figure 4 and Figure S2). The significant decline in proline accumulation, a well-characterized osmoprotectant critical for cellular osmotic adjustment under abiotic stress, indicates compromised osmoregulatory capacity and diminished tolerance to pH stress (Figure 2). This metabolic perturbation is mechanistically linked to the rapid accumulation of ROS in root tissues and the consequent oxidative damage to cellular macromolecules (Figure 2 and Figure 5). Although ROS serve essential roles as redox signaling molecules during adaptive stress responses, sustained or excessive ROS generation overwhelms endogenous antioxidant systems, leading to irreversible oxidative damage and amplification of stress-induced physiological dysfunction [9].

Peroxidase system homeostasis is tightly coupled with cellular ROS dynamics in plants. In this study, we observed a significant increase in SOD activity, no significant change in CAT activity, and a marked decline in POD activity under pH stress (Figure 6). These alterations in enzymatic activity indicate that the antioxidant defense response, though partially activated via SOD upregulation, fails to fully counteract ROS accumulation, resulting in oxidative damage to cellular components. Consistent with this, elevated MDA content, a well-established biomarker of membrane lipid peroxidation, provides biochemical confirmation of oxidative damage in seedlings subjected to acidic or alkaline pH stress (Figure 2). Notably, exogenous IAA priming effectively mitigated pH stress-induced phytotoxicity, as evidenced by significant recovery of root and hypocotyl elongation (Figure 10). Consistent with these observations, exogenous IAA application enhances the antioxidant enzyme activity, including CAT, POD, and SOD, in rice, thereby suppressing ROS and MDA accumulation and alleviating alkaline stress-induced phytotoxicity [14]. A substantial body of evidence demonstrates that bolstering endogenous ROS-scavenging capacity, through transcriptional and post-translational modulation of antioxidant systems, is a central mechanism underpinning alkaline stress tolerance in plants [8,9]. Similarly, auxin improves cucumber resilience to alkaline stress by coordinately upregulating antioxidant enzyme expression and enhancing cellular ROS detoxification efficiency [37]. In maize, IAA seed priming confers salinity tolerance by stimulating endogenous IAA biosynthesis and augmenting antioxidant enzyme activities, leading to enhanced ROS scavenging and reduced oxidative damage [38]. Exogenous melatonin application strengthens the plant antioxidant defense system, thereby enhancing drought tolerance in wheat and maize [33,34,39]. Similarly, IAA seed priming promotes cotton germination and early seedling vigor by coordinately regulating endogenous phytohormone homeostasis and sucrose metabolic flux [40]. Building upon this mechanistic evidence, we hypothesize that exogenous IAA application mitigates abiotic stress-induced injury in tomato through dual modulation of auxin homeostasis and antioxidant capacity.

Plant cell expansion and elongation are physically constrained by the load-bearing cell wall; its dynamic remodeling, underpinned by cycles of controlled loosening and re-stiffening, is orchestrated by EXP and XTHs. XTHs constitute a central class of cell wall-modifying enzymes that catalyze the selective cleavage and transglycosylation of xyloglucan polymers, thereby enabling cellulose microfibril rearrangement [41]. In contrast, EXPs are non-enzymatic proteins that induce cell wall stress relaxation by disrupting non-covalent bonds between cellulose and matrix polysaccharides, facilitating irreversible wall creep and cellular extension [42]. Consistent with these mechanistic roles, transcriptional expression analysis revealed significant downregulation of genes encoding key cell wall remodeling proteins under acid-base stress, including multiple *XTH* isoforms and *EXP* family members, particularly those associated with cell turgor-driven swelling and longitudinal elongation (Figure 9). Functional studies demonstrate that *SlEXP4* promotes tomato seed germination by facilitating endosperm cap weakening through targeted cell wall depolymerization [43]. In soybean, *GmEXP1* is essential for both initiation and sustained elongation of the primary root system [44]. Similarly, maize expansin isoforms *ZmEXP1*, *ZmEXP5*, *ZmEXP6*, and *ZmEXP8* collectively drive longitudinal root cell expansion [45]. Moreover, comparative transcriptomic analyses reveal that drought stress induces differential expression of multiple expansin genes in roots, and transgenic overexpression of specific expansins enhances root architectural plasticity and confers improved drought resilience, highlighting their functional contribution to abiotic stress adaptation [45,46]. Collectively, these findings indicate that acid-base stress-induced downregulation of *XTH* and *EXP* gene expression impairs cell wall remodeling capacity, thereby inhibiting root elongation. Exogenous IAA application effectively alleviates this inhibition and restores root growth under pH stress. Substantial evidence demonstrates that auxin orchestrates cell elongation by modulating the expression and activity of cell wall-modifying proteins, including EXPs and XTHs, thereby facilitating controlled wall loosening and structural reorganization [47,48,49,50]. Accordingly, we propose that auxin mediates root and hypocotyl elongation recovery under acid-base stress, at least in part, through transcriptional reactivation of key cell wall extension genes.

Moreover, chlorophyll a and b contents were significantly reduced in tomato seedlings subjected to acidic stress (pH 3.5), whereas chlorophyll a and carotenoid levels were markedly diminished under alkaline stress (pH 9.5) (Figure S1C–F). These stress-specific pigment perturbations suggest distinct underlying regulatory mechanisms, likely reflecting differential impacts of H^+^ excess versus OH^−^ excess on chloroplast development, thylakoid membrane integrity, and pigment biosynthesis pathways. Critically, reductions in photosynthetic pigment content correlate strongly with impaired photosynthetic performance, resulting in diminished carbon assimilation and consequent suppression of biomass accumulation [14]. Consistent with this, the observed declines in chlorophyll and carotenoid levels under both pH extremes align mechanistically with the significant reduction in seedling fresh weight (Figure S1), indicating compromised photoautotrophic capacity. Exogenous IAA seed priming significantly promoted both root and hypocotyl elongation in tomato seedlings under alkaline stress (pH 9.5) (Figure 10C,D). In contrast, under acidic stress (pH 3.5), IAA treatment enhanced hypocotyl elongation but exerted no statistically significant effect on root growth (Figure 10C,D). Consistent with these observations, IAA application improves rice growth under alkaline stress by transcriptionally upregulating auxin biosynthesis genes (such as *YUC* and *TAR*), thereby counteracting stress-induced growth inhibition [14]. Furthermore, IAA seed priming sustains endogenous IAA homeostasis during early seedling development and synergistically modulates germination and post-germinative growth through crosstalk with GA, JA, and ABA [38]. Collectively, these findings demonstrate that exogenous IAA priming is an effective strategy to mitigate alkaline stress-induced growth impairment in plants.

Differential expression of the *YUCs* under acidic and alkaline stress implies distinct mechanisms underlying auxin-mediated regulation of plant responses to pH stress. Consistent with this, exogenous IAA application differentially alleviated inhibition of tomato seed germination and seedling growth induced by acidic versus alkaline conditions. Specifically, the mitigating effect of IAA was more pronounced under alkaline stress (pH 9.5) than under acidic stress (pH 3.5). Two non-mutually exclusive explanations may account for this asymmetry. First, the narrow concentration range of IAA tested may have precluded identification of an optimal dose for maximal mitigation under acidic conditions. Second, the physiological impacts of low-pH and high-pH environments differ fundamentally: acidic conditions elevate proton (H^+^) activity, promoting solubilization and bioavailability of certain metal ions, whereas alkaline conditions increase OH^−^ concentration, leading to precipitation and reduced bioavailability of essential micronutrients such as Fe, Zn, and Mn [51,52]. These contrasting ionic and nutrient dynamics likely engage divergent signaling and homeostatic pathways, potentially influencing IAA stability, transport, conjugation, or perception. Nevertheless, IAA priming ameliorated extreme pH-induced germination and early seedling growth inhibition, confirming auxin’s functional involvement in the integrated regulatory network governing plant adaptation to both acidic and alkaline stress.

This study elucidates the integrated physiological and molecular mechanisms underlying plant seed germination and seedling growth inhibition under acidic and alkaline stress, with particular emphasis on auxin signaling, ROS homeostasis, and cell wall remodeling. Our findings establish that perturbations in these three interconnected regulatory modules (auxin biosynthesis, ROS scavenging capacity, and EXPs/XTHs-mediated wall loosening) are central to impaired germination, aberrant seedling morphogenesis, and suppressed vegetative growth under pH stress. Future work should prioritize functional characterization of key candidate genes (e.g., *YUCs*, *SOD*, *POD*, *XTHs*, *EXPs*) identified herein using targeted gene editing approaches, enabling the rational development of crop varieties with enhanced tolerance to acidic or alkaline soils. Moreover, translational strategies, including precise modulation of auxin metabolism, ROS buffering systems, and cell wall extensibility, hold significant promise for improving field performance of major crops in pH-stressed agroecosystems. Complementary agronomic interventions, such as selection and deployment of native acid or alkali-tolerant cultivars, optimized crop rotation systems, judicious application of organic amendments, and integrated soil ecological restoration, will collectively enhance soil health, sustain productivity, and ensure food security in marginal pH-affected farmlands.

## 4. Materials and Methods

### 4.1. Plant Materials and Cultivation

*Solanum lycopersicum* cv. Micro-Tom and the *DR5:GUS* reporter line were used in this study. Tomato seeds were plated on half-strength Murashige and Skoog medium (Duchefa Biochemie, Haarlem, The Netherlands) supplemented with 0.8% (*w*/*v*) agar. Plates were incubated vertically in a controlled environment growth chamber maintained at 25 °C under a 14 h light/10 h dark cycle.

### 4.2. Seed Germination Assay

Tomato seeds were placed on double-layered filter paper in 9 cm diameter Petri dishes, and 10 mL of solutions with the respective pH gradients were added to each dish. Solutions spanned an eight-point pH gradient: three acidic treatments (pH 3.5, 4.5, 5.5), a near-neutral control (pH 6.5), and four alkaline treatments (pH 7.5, 8.5, 9.5, 10.5). All solutions were prepared in sterile distilled water and pH-adjusted to target values using 1 M HCl or KOH. Dishes were incubated in a climate chamber. Germination was scored daily as radicle emergence (>2 mm). To maintain pH stability, treatment solutions were refreshed every 48 h.

Seed priming with IAA: Tomato seeds were immersed in a 10 µM IAA solution for 24 h. Subsequently, the imbibed seeds were transferred to three liquid culture solutions adjusted to pH 3.5, 6.5, and 9.5, respectively, and incubated under standardized germination conditions. Germination was monitored daily, and root and hypocotyl length were measured at the conclusion of the 7-day experiment. To maintain stable pH throughout the treatment period, the solution was refreshed every 48 h.

### 4.3. Cell Viability Assays

Cell viability in seeds and seedling roots was assessed using the 2,3,5-triphenyltetrazolium chloride (TTC) reduction assay. For seed assays, tomato seeds were subjected to 48 h treatments under pH 3.5, 6.5, or 9.5 conditions, respectively. Following treatment, seeds were rinsed with sterile distilled water, manually decorticated, and incubated in 1% (*w*/*v*) TTC solution for 2 h. For seedling root assays, three-day-old tomato seedlings were transferred to the solution (pH 3.5, 6.5, or 9.5) and incubated for 12 h. Subsequently, the samples were incubated in 0.5% (*w*/*v*) TTC solution for 30 min. Tomato seedlings were incubated separately in 0.25% (*w*/*v*) Evans blue or 0.4% (*w*/*v*) trypan blue solution for 15 min. Images were acquired under a stereomicroscope (SZN71, Sunny Instruments Co., Ltd., Ningbo, China).

### 4.4. Detection of ROS

H_2_O_2_ and O_2_^∙−^ accumulation in tomato seedling roots were visualized using 3,3′-diaminobenzidine (DAB) and nitroblue tetrazolium chloride (NBT) histochemical staining, respectively, according to Wei et al. (2024) [53]. Three-day-old tomato seedlings were transferred to the treated solution (pH 3.5, 6.5, or 9.5) and incubated for 6 h. Following treatment, roots were immersed in 0.25% (*w*/*v*) NBT or 1 mg·mL^−1^ DAB.

### 4.5. GUS Histochemical Staining

GUS activity was assayed as described previously [54]. Tomato seeds or three-day-old tomato seedlings were transferred to a pH condition (pH 3.5, 6.5, or 9.5) and incubated for 48 h. The samples were rinsed with ddH_2_O and immersed in GUS solution for 24 h. Subsequently, the seeds and seedlings were rinsed briefly with distilled water and imaged under a stereomicroscope (Nikon TS2-FL, Tokyo, Japan).

### 4.6. Antioxidant Enzyme Activity Assays

Antioxidant enzyme activities were assayed by the method described previously [55]. Crude enzyme extracts were prepared from roots of tomato seedlings subjected to pH treatments (pH 3.5, 6.5, or 9.5). Approximately 0.3 g of fresh weight of roots was homogenized in 1 mL phosphate buffer. The sample was rinsed with another 2 mL of the same buffer, transferred to a microcentrifuge tube, and centrifuged at 12,000× *g* for 15 min. The supernatant was harvested as crude enzyme extract and maintained on ice for subsequent spectrophotometric measurement of SOD, CAT, and POD activities.

### 4.7. Determination of Malondialdehyde, Proline, and Photosynthetic Pigment Contents

Tomato seedlings subjected to pH treatments (pH 3.5, 6.5, or 9.5) for 7 days were harvested for physiological and biochemical analyses. For each treatment, 0.5 g of fresh plant seedlings was harvested and separately analyzed for malondialdehyde (MDA) and free proline contents. MDA and free proline contents were quantified using the 2-thiobarbituric acid (TBA) assay and the acid-ninhydrin colorimetric method, respectively, according to the previously described method [56].

Photosynthetic pigment contents were determined using the ethanol extraction method as previously described [57]. Briefly, 0.1 g of tomato seedling leaves was homogenized in 5 mL 95% (*v*/*v*) ethanol. After filtering, the supernatant was analyzed using a spectrophotometer. Sample absorbance was determined at wavelengths of 665, 649, and 470 nm.

### 4.8. Gene Expression Analysis

Total RNA was isolated from roots of tomato seedlings exposed to pH treatments (pH 3.5, 6.5, and 9.5) using TRIzol reagent, according to the instructions for the RNA Extraction Kit (RE600) (Coolaber, Beijing, China). RNA quality was assessed via a UPT200 ultramicro spectrophotometer (UNICO, Shanghai, China), and first-strand cDNA was synthesized from 1 μg of total RNA using the reverse transcription kit RM201-100T (Coolaber, Beijing, China). Quantitative PCR was performed on a LightCycler 480 II system (Roche, Basel, Switzerland) with Universal SYBR qPCR Mix (Tsingke Bio., Nanjing, China). SlUBQ served as the internal reference gene.

### 4.9. Statistical Analysis

Data were organized and visualized using Excel 2019, and statistical analyses were conducted with PASW Statistics 18. Statistical significance was determined by Student’s *t*-test or one-way ANOVA. Data presented as means ± SD. For germination assays, 30 tomato seeds were assigned to each treatment. For physiological and biochemical analyses, 15–30 uniform tomato seedlings were sampled per treatment.

## 5. Conclusions

**Figure 12:**
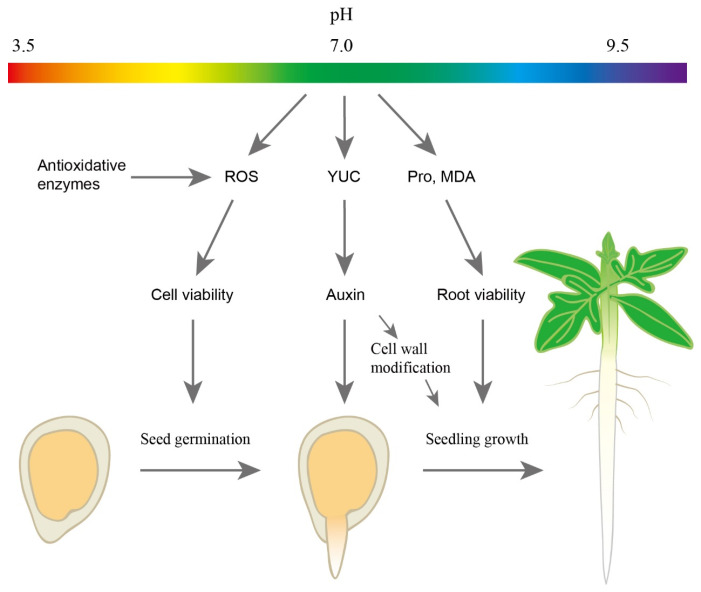
A proposed regulatory model of tomato seed germination and seedling growth under pH stress mediated by auxin and ROS. Under neutral pH conditions, endogenous ROS levels are maintained within physiological ranges through balanced antioxidant enzyme activity, ensuring redox homeostasis without cellular damage to seeds or seedlings. In contrast, exposure to acidic or alkaline stress disrupts this equilibrium; excessive ROS accumulation overwhelms the antioxidant system, leading to oxidative damage, diminished cell viability, and impaired membrane integrity (MDA). A decrease in proline content further exacerbates the detrimental impacts of pH stress in plants. Furthermore, acid-base stress reduces auxin levels in plants by downregulating the auxin synthesis gene *YUC* expression. This disrupts auxin-mediated cell wall modification and remodeling, thereby inhibiting cell enlargement and primary root elongation. The synergistic disruption of redox balance and auxin signaling cascades underlies the inhibition of seed germination and early seedling establishment in tomato under non-optimal pH conditions.

This study systematically elucidates the physiological and molecular mechanisms underlying tomato seed germination inhibition and seedling growth suppression under pH stress, integrating phenotypic, biochemical, hormonal, and transcriptional expression of genes across a gradient of pH treatments (Figure 12). Acidic (pH 3.5) and alkaline (pH 9.5) stress each triggered rapid accumulation of ROS, impaired antioxidant enzyme system homeostasis, and consequently induced severe lipid peroxidation (elevated MDA content), culminating in diminished cell viability. Concurrently, endogenous auxin levels were significantly depleted in the radicle and primary roots, reflecting disrupted auxin biosynthesis. Gene expression analysis further revealed coordinated downregulation of key cell wall remodeling genes including *EXPs* and *XTHs*, thereby compromising cell wall extensibility essential for radicle emergence and hypocotyl elongation. Moreover, proline accumulation was markedly attenuated under both pH extremes, diminishing osmoprotective capacity and exacerbating oxidative and growth defects. Collectively, these data identify the synergistic disruption of ROS homeostasis, auxin signaling, and proline-mediated osmotic adjustment, as well as consequent impairment of cell wall dynamics, as the core determinants of reduced seed viability and arrested seedling development under pH stress. Critically, IAA seed priming effectively restored IAA homeostasis and significantly improved germination rates and early seedling vigor under both acidic and alkaline conditions. These findings provide mechanistic insight into plant pH stress responses and establish auxin modulation as a physiologically grounded strategy to enhance crop resilience in acid- or alkali-affected soils.

## Data Availability

Data are contained within the article.

## Supplementary Materials

The following supporting information can be downloaded at: https://www.mdpi.com/article/10.3390/plants15132017/s1, Figure S1: Fresh weight, lateral root development, and photosynthetic pigment content of tomato seedlings under pH gradient conditions; Figure S2: Effects of acidic or alkaline stress on cell death in seedling roots; Figure S3: IAA alleviated ROS accumulation triggered by alkaline pH stress; Figure S4: Effect of IAA on antioxidant enzyme activity in tomato seedlings; Table S1: Primer list used in the study. Reference [58] is cited in Supplementary Materials.
