# Supplementary material for: Acidic and Alkaline pH Stresses Impair Tomato Seed Germination and Seedling Growth via Disruption of Reactive Oxygen Species and Auxin Homeostasis
Source: Plants (Basel). 2026 Jun 29;15(13):2017. doi: 10.3390/plants15132017 (PMC13364135; doi:10.3390/plants15132017)
Supplement: Supplementary file 1 [file plants-15-02017-s001.zip › plants-4331658-supplementary.pdf]

Supplementary Materials:

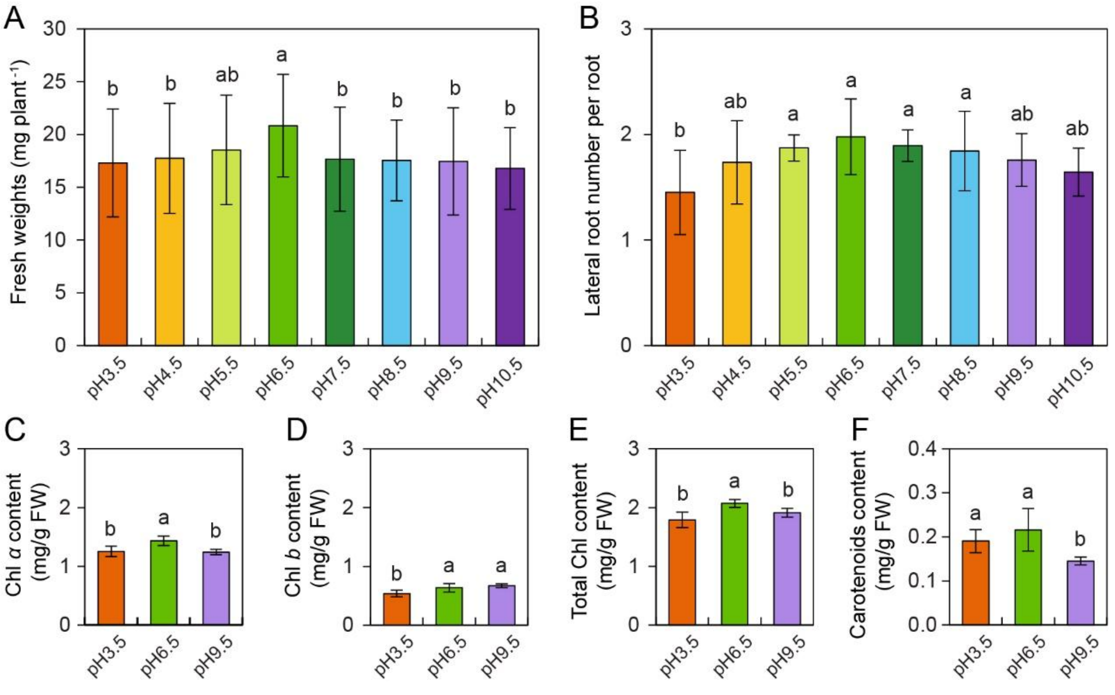

Figure S1. Fresh weight, lateral root development, and photosynthetic pigment content of tomato seedlings under pH gradient conditions. (A, B) Fresh weight (A) and lateral root number (B) of tomato seedlings at 7 days post-germination under pH gradient conditions (pH 3.5, 4.5, 5.5, 6.5, 7.5, 8.5, 9.5, and 10.5). (C–F) Chlorophyll a (Chl *a*), chlorophyll b (Chl *b*), total chlorophyll (Chl), and carotenoid content in leaves of 7-day-old tomato seedlings exposed to pH 3.5, 6.5, or 9.5. Data are means ± SD. Columns followed by different letters are significantly different at $P < 0.05$.

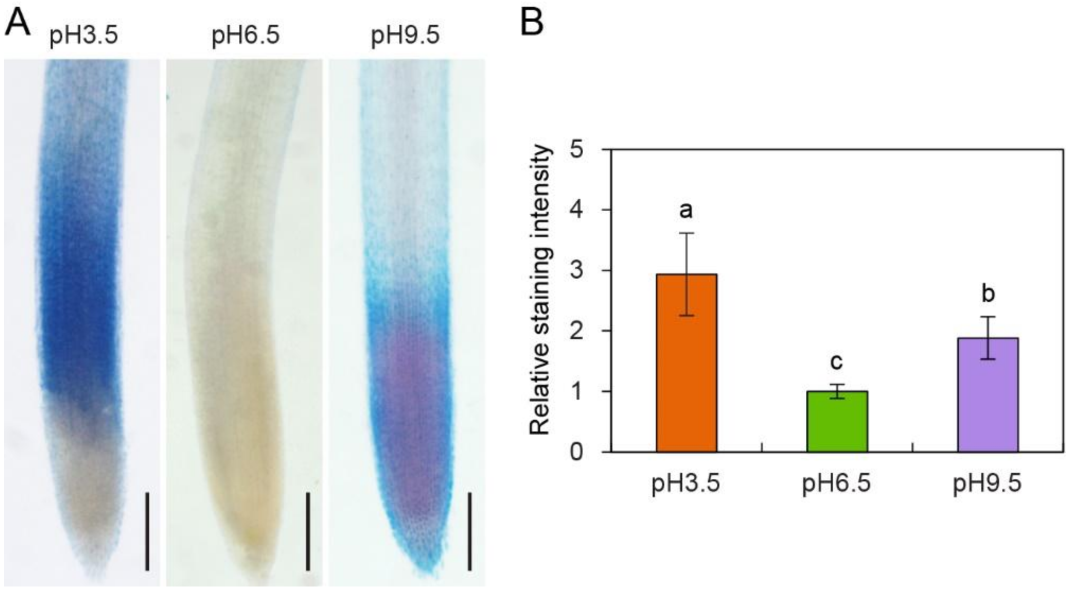

Figure S2. Effects of acid or alkaline stress on cell death in seedling roots. Three-day-old tomato seedlings were exposed to acidic (pH 3.5), alkaline (pH 9.5), or control (pH 6.5) conditions for 12 h. Cell death was assessed using trypan blue staining (A and B). Data are means ± SD. Columns followed by different letters are significantly different at $P < 0.05$. Bars = 200 μm.

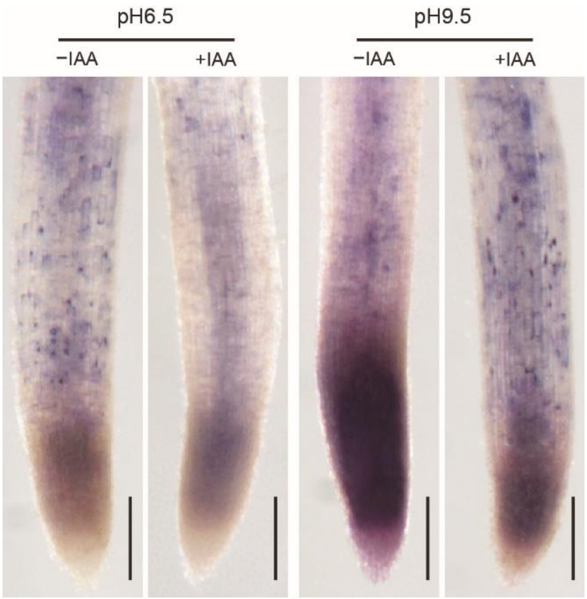

Figure S3. IAA alleviated ROS accumulation triggered by alkaline pH stress. Tomato seeds were pretreated with 10 μM IAA and germinated under pH 6.5 or 9.5 conditions for 5 days. ROS accumulation in tomato seedling roots was detected using NBT staining.

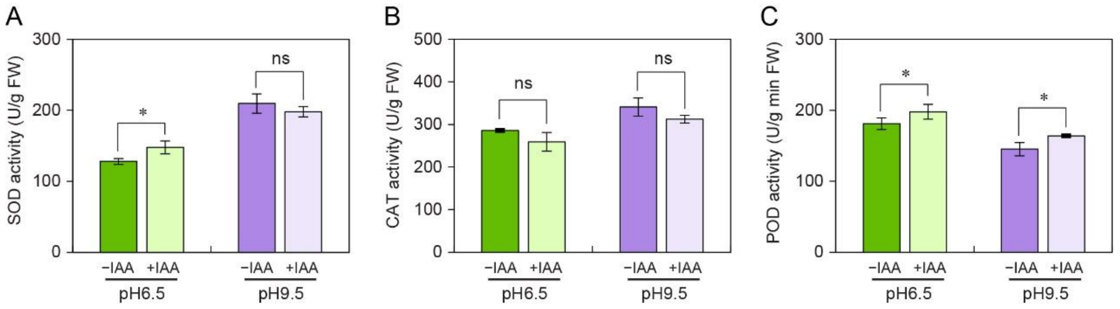

Figure S4. Effect of IAA on antioxidant enzyme activity in tomato seedlings. (A-C) SOD (A), CAT (B), and POD (C) activities of tomato seedlings at 5 days post-germination under alkaline (pH 9.5) or control (pH 6.5) conditions. Tomato seeds were pretreated with 10 μM IAA and germinated under pH 6.5 and 9.5 conditions. Data are means ± SD. Asterisks indicate that values between IAA treatments at each pH level differ significantly (*$p < 0.05$). ns represents not significant.

Table S1. Primer list used in the study

Primer	Forward primer (5' to 3')	Reverse primer (5' to 3')
SlXTH2	ATGGGGTTATGGGAGGGTGA	TGCCAGCAGAATTACCAGGG
SlXTH4	TTGGGCTACAAGAGGAGGGT	CTCTTGTGGTGTGACAGCCT
SlXTH5	GGGATGCTTCAGATTGGGCT	AATCTCGCACTCTGGTGGTG
SlXTH9	CGTCTGGAATCCTCATCGCA	GTGCAAGTGCCCAATATTCCA
SlEXP1	AATGGTGGCTGGTGTAACCC	ATTCCTCCTTGCTTTCGGCA
SlEXP5	CCTCTATTTATTCTCCACGATTGCT	TGTTCGTTCCGTATCCCGTG
SlEXP6	ACTACCGAAGGGTTCCATGC	CATGGCGTGTTTGTGCCTTT
SlEXP8	TGCAAGAAGCATGGTGGAGT	ATTGCCAACTTGCTCCCCAA
SlEXP11	TCATCTACTTTTCTTGGCTTAGACC	TTCCCATAGCCACAAGCACC
SlUBQ	CACCAAGCCAAAGAAGATCA	TCAGCATTAGGGCACTCCTT

Primer sequences (*SlYUC1*–*SlYUC9*) were referenced from previous report [58].
